# Metabolic Power in Team and Racquet Sports: A Systematic Review with Best-Evidence Synthesis

**DOI:** 10.1186/s40798-022-00525-9

**Published:** 2022-10-25

**Authors:** Joana Brochhagen, Matthias Wilhelm Hoppe

**Affiliations:** Movement and Training Science, Leipzig University, Jahnallee 59, 04109 Leipzig, Germany

**Keywords:** Energetic demand, Energy expenditure, Energy supply, Football, Global positioning system, Heart rate, Internal load, Local positioning system, Soccer, Video camera system

## Abstract

**Background:**

In intermittent team and racquet sports, metabolic loads are rarely investigated as they are difficult to examine, e.g., by portable metabolic carts and lactate measures. However, determining the instantaneous metabolic power of intermittent running from acceleration and speed data is possible. Recently, this potential has gained more interest in research and practice due to the development of player tracking technologies that allow easy access to the required data. The aim of this review was to systematically investigate the validity and point out the evidence of this new approach for estimating metabolic loads in intermittent sports. To provide an in-depth understanding of this approach and its validity, the fundamental aspects of the underlying concept were also considered.

**Methods:**

PubMed®, Cochrane Library, Web of Science™, and BISp-surf databases were included in the search conducted on March 1, 2021. Studies assessing physiological and methodological validation as well as conceptual studies of the metabolic power approach in intermittent sports players without diseases or injuries were deemed eligible. The quality assessment was implemented using a modified 12-item version of the Downs and Black checklist. Additionally, a best-evidence synthesis of the validation studies was performed to clarify the direction and strength of the evidence.

**Results:**

Of 947 studies that were identified, 31 met the eligibility criteria of which 7 were physiological, 13 methodological validation, and 11 conceptual studies. Gold standards for validating the metabolic power approach were predominantly oxygen uptake with 6 and traditional running speed analysis with 8 studies for physiological and methodological validation, respectively. The best-evidence synthesis showed conflicting to strong and moderate to strong evidence for physiological and methodological validity of the approach, respectively. The conceptual studies revealed several modifications regarding the approach that need to be considered. Otherwise, incorrect implementation can occur.

**Conclusions:**

Evidence of the physiological validity of the metabolic power approach ranged from conflicting to strong. However, this should be treated with caution as the validation studies were often partially implemented incorrectly as shown by the underlying concept studies. Moreover, strong evidence indicated that the approach is valid from a methodological perspective. Future studies must consider what the metabolic power approach can and cannot actually display.

## Key Points

- A lack of research exists in studies concerning children, females, and team and racquet sports besides soccer and the application of more profound physiological approaches for the validation and assessment of metabolic power estimated by acceleration and speed data is needed.
- Previous physiological validation studies are outdated as there have been adaptations concerning the metabolic power approach for estimating metabolic loads over recent years, and methodological validation studies revealing its superiority over the traditional running speed approach.
- Distinction between walking and running, different terrains, as well as aerobic and anaerobic energy supply should be considered when assessing metabolic power in team and racquet sports.

## Introduction

Match and training demands of athletes can be described by external and internal loads [1]. In particular, internal loads are important to monitor, for example, with respect to neuronal, cardiovascular, metabolic, and hormonal stimuli because they are involved in regulating the gene expression required for all regeneration and adaptation processes [2–4]. In team and racquet sports, which are characterized by an intermittent activity profile [5, 6], cardiovascular loads are well investigated [7]. However, less is known about metabolic loads as they are more difficult to examine from a methodological point of view [8]. Standard procedures to assess metabolic loads are based on body temperature, heart rate, oxygen uptake, and lactate measures [3]. More advanced procedures include the measurement of creatine phosphate concentration [9] or the use of chemical isotopes as doubly labeled water [10]. However, these procedures are partially invasive and poorly reproducible [11, 12], difficult to apply during matches or training [3, 13], do not allow real-time monitoring [14], and do not fully and continuously scope the metabolic loads in intermittent sports [14]. Therefore, new methodological approaches to assess metabolic loads in a valid and practical manner for intermittent sports are needed.

In 2005, di Prampero et al. [15] suggested a solution where it is possible to determine the instantaneous metabolic power of accelerated running. Metabolic power describes the amount of energy needed to maintain a constant ATP level [16]. The approach is based on the extrapolation from the external (mechanical) to the internal (metabolic) load, for which two assumptions must be considered: (1) accelerated running on a flat terrain is energetically equivalent to running up a slope at constant speed and (2) the relative energy cost for running is independent of the speed and amounts to approximately 3.6–4.0 J/kg/m [17, 18]. Based on these assumptions, the relative energy cost for accelerated running on a flat terrain can be estimated. The subsequent multiplication with the underlying speed leads to the instantaneous metabolic power in W/kg [19]. The original equations are as follows:

1 $$\mathrm{EC}=\left(155.4{\mathrm{ES}}^{5}-30.4{\mathrm{ES}}^{4}-43.3{\mathrm{ES}}^{3}+46.3{\mathrm{ES}}^{2}+19.5\mathrm{ES}+3.6\right)\mathrm{EM}$$

2 $$P=\mathrm{EC}v$$

where EC is the energy cost, ES the equivalent slope, 3.6 the relative energy cost for running at constant speed, EM the equivalent mass, P the metabolic power, and v the speed. Knowledge of the metabolic power, calculated from acceleration and speed data, can be of value when investigating energetic match demands and assessing training loads in intermittent sports [19].

Even though the approach was published in 2005 [15], it has recently gained greater interest in research and practice. This may be caused by the development of several player tracking technologies, such as global (GPS) and local positioning systems (LPS), allowing easy [20] and accurate [21] access to the required acceleration and speed data [20]. However, in order to apply such an innovative metabolic approach into research and practice, it is necessary to clarify its validity on both a physiological and methodological level for which appropriate gold standards are essential [19]. A key aspect for validation purposes of the metabolic power approach is an in-depth understanding of the underlying concept, which has been described by a few narrative reviews [8, 19]. To date, there is no systematic review of the metabolic power approach in intermittent sports that clarifies the validity as the most important quality criterion of quantitative research [22]. In general, its strength and direction are prerequisites for providing trustworthy, consistent, neutral, and practical-applicable evidence [22, 23]. Therefore, the aim of this review was to systematically investigate the validity and point out the evidence of the metabolic power approach for estimating metabolic loads in intermittent team and racquet sports.

## Methods

### Research Design and Search Strategy

The Preferred Reporting Items for Systematic Reviews and Meta-Analyses (PRISMA) statement was applied [24]. The literature search was conducted in English on March 1, 2021, and included the following four databases: PubMed®, Cochrane Library, Web of Science™, and BISp-surf. The search keywords were divided into components using the PICO scheme (P = Population, I = Intervention, C = Comparisons, and O = Outcomes) [24]. The components were as follows: P = Intermittent sports players without diseases or injuries; I = Tracking; C = Metabolic power approach by di Prampero et al. [15]; and O = Metabolic loads. The component for Comparisons (C) was excluded from the search line as it would have resulted in studies authored by di Prampero only. The resulting final search line was applied to all fields of the database search and was as follows: (team sport OR field sport OR racquet sport OR soccer OR football OR hockey OR rugby OR handball OR volleyball OR basketball OR lacrosse OR futsal OR tennis OR table tennis OR badminton) AND (tracking technology OR global positioning system OR local positioning system OR video camera system OR speed OR acceleration OR deceleration) AND (metabolic power OR energy cost OR energy expenditure). The identified entries were downloaded to a citation manager (Clarivate Analytics, EndNote X9.2, London, UK) and duplicates were removed. The remaining studies were transferred to a spreadsheet (Microsoft Office, Excel 2016, Redmond, USA). First, titles and abstracts followed by full texts were screened for eligibility criteria and studies deemed unsuitable were removed. Additionally, a secondary search based on the reference lists of the studies deemed eligible was conducted. All methodological procedures were executed independently by two researchers (JB, AS) and in case of any disagreement a third (MWH) made the decision.

### Eligibility Criteria

The eligibility criteria were set and agreed on by both authors. The criteria for screening titles and abstracts were as follows:

1. Published in 2005 or later (as the original metabolic power approach was introduced in 2005);
2. Written in English;
3. Not systematic review; and
4. Topic on intermittent sports, metabolic power, no animals, no diseases, and no injuries.

The criteria for full texts were as follows:

1. Written in English;
2. Topic on the metabolic power approach by di Prampero et al. [15]; and
3. Either a physiological or methodological validation or conceptual study.

### Quality Assessment

The quality assessment was implemented using a modified version of the Downs and Black checklist [20]. Briefly, 12 of the original 27 criteria were used. Original questions 5, 8, 9, 13–15, 17, 19, and 21–27 were removed as they were not suitable regarding the purpose of the study. For questions 3, 7, 10–12, and 18 “not applicable” was used as a fourth scoring option. These modifications were conducted especially due to the conceptual studies often being based on a theoretical approach without the inclusion of subjects as well as the absence of statistical analyses. To account for the non-medical purpose of the present review, further modifications were made concerning the terms used as “patient” was replaced with “subject,” “intervention” with “condition” and “treatment” with “testing,” as previously done [25]. The final quality score for a study—after excluding the questions marked as “not applicable”—was expressed as a percentage. Hence, a higher percentage shows a higher quality of a study regarding the applied quality assessment procedure. The rating of the studies was as follows: low (≤ 33.3%); moderate (33.4–66.7%); and high (≥ 66.8%) quality, as previously recommended [26].

### Data Extraction

Data of the physiological and methodological validation and conceptual studies were extracted based on the PICO scheme by one researcher (JB). Thereby, the following items were presented (if applicable): (1) P = Type of sport, number of participants, sex, age, playing level, and nationality; (2) I = Setting of the study, tests, and matches; (3) C = Aim or gold standard used for validation; and (4) O = Main results.

### Synthesis of Results

To further clarify the results of the data extraction concerning the direction and strength of evidence for the validation studies, a best-evidence synthesis was performed for which previously defined criteria were used (Table 1) [27]. Minor modifications were made regarding the terms used to describe the study quality: “acceptable” was replaced with “moderate” and “borderline” with “low” to account for the quality ratings of the applied Downs and Black checklist.

**Table 1:** Criteria for the best-evidence synthesis according to Asker et al. [27]

Rating	Study quality	Criterion
Strong evidence	≥ 2 high-quality studies	≥ 75% consistent findings in these studies
Moderate evidence	1 high-quality study and/or ≥ 2 moderate quality studies	≥ 75% consistent findings in these studies
Limited evidence	1 moderate quality study and/or ≥ 1 low-quality studies	/
Conflicting evidence	≥ 2 studies of any quality	< 75% consistent findings in these studies

## Results

### Literature Search

**Fig. 1:**
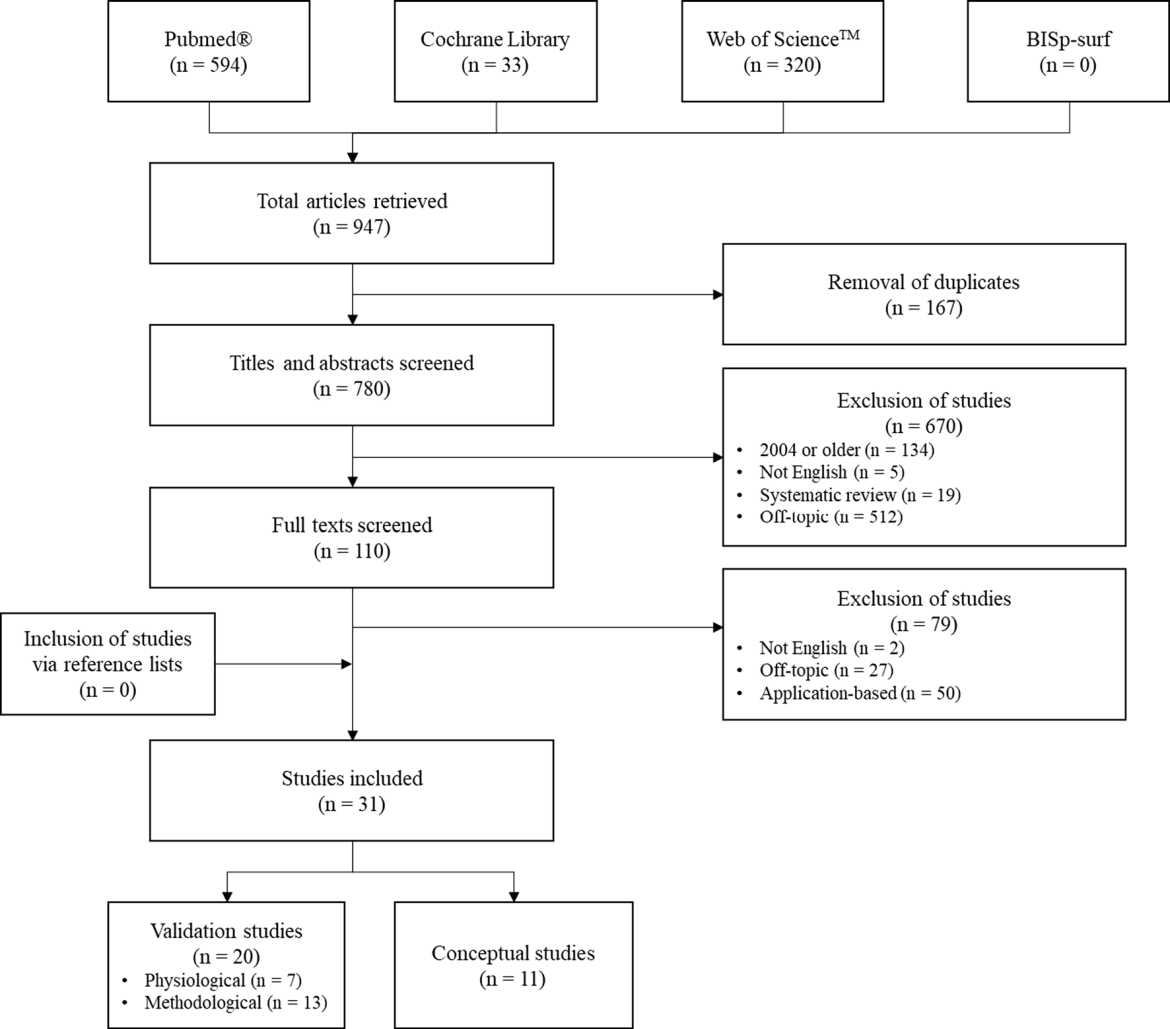
Flowchart of the literature search including the study selection process according to the PRISMA guidelines

Figure 1 shows the results of the literature search. In total, 947 studies were found. After the removal of 167 duplicates, the titles and abstracts of the remaining 780 studies were screened. Out of these, 670 studies were removed because they did not meet the eligibility criteria, leaving 110 studies for screening of the full text. By screening the full texts, another 79 studies were excluded based on the eligibility criteria. No further studies were eligible via the reference lists. Finally, 31 studies were included. Twenty of these studies were validation studies from which 7 and 13 used physiological [28–34] and methodological approaches [35–47], respectively. The remaining 11 studies were conceptual studies [3, 8, 16, 17, 19, 48–53].

### Quality Assessment

Table 2 presents the results of the quality assessment. The mean quality score of all studies was 78.6%. The corresponding scores for physiological and methodological validation as well as conceptual studies were 77.4, 75.6, and 82.8%, respectively. Questions 3, 6, 7, 8, 9, and 11 were partially “not applicable” for 4 conceptual studies [16, 17, 19, 50]. Questions 1, 2, 4, 10, and 12 were scored “yes” for all studies. Additionally, questions 3 and 11 were scored “yes” for all validation studies and the latter for all applicable conceptual studies. For all validation and applicable conceptual studies, question 8 was rated “unable to determine” and 9 “no.”

**Table 2:** Quality assessment of the validation and conceptual studies using the Downs and Black checklist

	Study (Year)	Criterion/Question												Score (%)	Quality
		1	2	3	4	5	6	7	8	9	10	11	12		
Physiological validation	Akubat et al. [28]	Y	Y	Y	Y	Y	Y	Y	U	N	Y	Y	Y	83.3	High
	Brown et al. [29]	Y	Y	Y	Y	Y	Y	N	U	N	Y	Y	Y	75.0	High
	Buchheit et al. [30]	Y	Y	Y	Y	Y	Y	Y	U	N	Y	Y	Y	83.3	High
	Highton et al. [31]	Y	Y	Y	Y	Y	Y	N	U	N	Y	Y	Y	75.0	High
	Manzi et al. [32]	Y	Y	Y	Y	Y	Y	N	U	N	Y	Y	Y	75.0	High
	Oxendale et al. [33]	Y	Y	Y	Y	Y	Y	N	U	N	Y	Y	Y	75.0	High
	Stevens et al. [34]	Y	Y	Y	Y	Y	Y	N	U	N	Y	Y	Y	75.0	High
														Mean: 77.4	
Methodological validation	Castagna et al. [35]	Y	Y	Y	Y	Y	Y	N	U	N	Y	Y	Y	75.0	High
	Darbellay et al. [36]	Y	Y	Y	Y	Y	Y	N	U	N	Y	Y	Y	75.0	High
	Dubois et al. [37]	Y	Y	Y	Y	Y	Y	Y	U	N	Y	Y	Y	83.3	High
	Gaudino et al. [38]	Y	Y	Y	Y	N	Y	N	U	N	Y	Y	Y	66.7	Moderate
	Gaudino et al. [39]	Y	Y	Y	Y	N	Y	N	U	N	Y	Y	Y	66.7	Moderate
	Goto and King [40]	Y	Y	Y	Y	Y	N	N	U	N	Y	Y	Y	66.7	Moderate
	Goto and Saward [41]	Y	Y	Y	Y	Y	Y	N	U	N	Y	Y	Y	75.0	High
	Hoppe et al. [42]	Y	Y	Y	Y	Y	Y	Y	U	N	Y	Y	Y	83.3	High
	Lord et al. [43]	Y	Y	Y	Y	Y	Y	Y	U	N	Y	Y	Y	83.3	High
	Lord et al. [44]	Y	Y	Y	Y	Y	Y	N	U	N	Y	Y	Y	75.0	High
	Martinez-Gabrera and Núnez-Sánchez [45]	Y	Y	Y	Y	Y	Y	N	U	N	Y	Y	Y	75.0	High
	Polglaze et al. [46]	Y	Y	Y	Y	Y	Y	Y	U	N	Y	Y	Y	83.3	High
	Scott et al. [47]	Y	Y	Y	Y	Y	Y	N	U	N	Y	Y	Y	75.0	High
														Mean: 75.6	
Conceptual studies	Di Prampero et al. [8]	Y	Y	N	Y	Y	Y	N	U	N	Y	Y	Y	66.7	Moderate
	Di Prampero and Osgnach [17]	Y	Y	N/A	Y	Y	Y	N/A	N/A	N/A	Y	N/A	Y	100.0	High
	Gaudino et al. [48]	Y	Y	Y	Y	Y	Y	N	U	N	Y	Y	Y	75.0	High
	Gray et al. [16]	Y	Y	N/A	Y	Y	N/A	N/A	N/A	N/A	Y	N/A	Y	100.0	High
	López-Fernández et al. [49]	Y	Y	Y	Y	Y	Y	Y	U	N	Y	Y	Y	83.3	High
	Osgnach et al. [3]	Y	Y	Y	Y	Y	Y	N	U	N	Y	Y	Y	75.0	High
	Osgnach and di Prampero [50]	Y	Y	N	Y	Y	N/A	N/A	N/A	N/A	Y	N/A	Y	85.7	High
	Polglaze and Hoppe [19]	Y	Y	N/A	Y	Y	N/A	N/A	N/A	N/A	Y	N/A	Y	100.0	High
	Ponzano and Gollin [51]	Y	Y	Y	Y	Y	Y	N	U	N	Y	Y	Y	75.0	High
	Savoia et al. [52]	Y	Y	Y	Y	Y	Y	N	U	N	Y	Y	Y	75.0	High
	Vescovi and Falenchuk [53]	Y	Y	N	Y	Y	Y	Y	U	N	Y	Y	Y	75.0	High
														Mean: 82.8	
														Total mean: 78.6	

*Y*  yes, *N*  no, *U*  unable to determine, *N/A*  not applicable

### Characteristics of the Studies

Table 3 summarizes the characteristics of the 7 physiological validation studies. Soccer was the most investigated sport with 4 studies [28, 30, 32, 34] followed by rugby with 1 study [31]. One study included different team sports (rugby, soccer, hockey, and netball) [33] and 1 study investigated undefined team sports [29]. The sex of the players was not specified in 3 studies [28, 30, 34]. Males [31, 32] and both sexes [29, 33] were studied in 2 studies each. Regarding age, 6 studies examined adult [28, 29, 31–34] and 1 study investigated youth players [30]. Three studies investigated amateurs [28, 29, 34], whereas professional [30, 32] and university players [31, 33] were examined in 2 studies, respectively.

**Table 3:** Characteristics and synthesis of the physiological validation studies using the PICO scheme

Study (Year)	Population	Intervention	Comparison	Outcome
Akubat et al. [28]	10 competitive amateur soccer players of unknown sex (20 ± 1 years)	2 test protocols at least 5 days apart: (1) lactate threshold test with 6 four-min stages (6, 8, 10, 12, 14, and 16 km/h) followed by a ramp test until exhaustion (increase of 0.2 km/h every 12 s); (2) modified version of Ball-Sport Endurance and Sprint Test for 30 min, performed twice with 2 days apart	Calculating iTRIMP and examine external/internal load ratios with GPS (5 Hz) derived metabolic power and PlayerLoad™; relationship between ratios and speed at lactate threshold and onset of blood lactate accumulation; influence of fatigue; use of modified equation for metabolic power analysis (Osgnach et al. [3])	Mean metabolic power ratio was largely correlated to speed at lactate threshold both in recovered (*r* = 0.59) and fatigued (*r* = 0.57) states; correlation to speed at onset of blood lactate accumulation was large (recovered, *r* = 0.61) and moderate (fatigued, *r* = 0.38); high metabolic power ratio was largely (recovered, *r* = 0.54) and small (fatigued, *r* = 0.27) correlated to speed at lactate threshold; correlation to speed at onset of blood lactate accumulation was large (recovered, *r* = 0.67) and very large (fatigued, *r* = 0.70)
Brown et al. [29]	27 team sport players (15 males, 12 females; 21 ± 2.7 years)	90 min exercise session on outdoor pitch divided into 6 bouts of 5 min of exercise (walking, jogging, running, 3 bouts of simulated team sport circuit) separated by 10 min of rest	GPS (5 Hz interpolated to 15 Hz) derived EE compared to VO_2_ (portable metabolic cart) derived EE; use of modified equation for metabolic power analysis (Osgnach et al. [3])	Moderate overall (complete 90 min session) underestimation of GPS derived EE (-19.0%); very large underestimation for team sport circuits (−44.0%); very large overestimation for walking (43.0%); no significant differences for jogging (7.8%) and running (4.8%)
Buchheit et al.[30]	14 French elite youth soccer players of unknown sex (15.4 ± 1.6 years)	4.5 min exercise circuit including technical actions with the ball (slaloms, pass and retrieve of a rebound wall, shot on goal) divided into 3 bouts of 1 min of exercise (at speeds of 6.5, 7.0, 7.5 km/h) separated by 30 s of rest; repetition of the circuit one week later	GPS (4 Hz) derived metabolic power compared to VO_2_ (portable metabolic cart) derived metabolic power; use of modified equation for metabolic power analysis (Osgnach et al. [3])	GPS derived metabolic power was 29 ± 10% lower during exercise and 85 ± 7% lower during recovery; correlation between GPS and VO_2_ derived metabolic power was small (*r* = 0.24, exercise and recovery phase) to moderate (*r* = 0.58, only exercise phase); reliability of GPS derived metabolic power was moderate (CV = 8.0%; ICC = 0.57)
Highton et al. [31]	16 male university rugby players (23.8 ± 4.8 years)	Repeated effort protocol to simulate physical contact including 3 sets of 6 rounds of: 8 m run at 14.4 km/h to collide with tackle bag to the ground, repositioning back, running backwards at 9 km/h to starting point	GPS (10 Hz) derived EE compared to VO_2_ (portable metabolic cart) derived EE; unknown equation for metabolic power analysis	GPS derived EE showed a systematic underestimation (−5.94 ± 0.67 kcal/min; ~ −45%); correlation between GPS and VO_2_ derived EE was moderate (*r* = 0.63)
Manzi et al. [32]	17 male professional Italian Serie A soccer players (28.2 ± 2.2 years)	Data from 19 championship matches and 2 aerobic fitness tests at least 24 h apart: (1) long-stage treadmill test for lactate profiling until exhaustion (1 km/h every 5 min until lactate of 4 mmol/l, then 0.5 km/h every 30 s); (2) short-stage running field test on a 400 m track for VO_2max_ until exhaustion (start 8 km/h and increase of 0.5 km/h every min)	Comparison of aerobic fitness variables (VO_2max_, VO_2_VT, %VO_2_VT, maximal aerobic speed, V_L4_) and match data in metabolic power categories (distance at high power: > 20 W/kg; very high power: > 35 W/kg; max power: > 55 W/kg) using a video camera system (25 Hz); use of modified equation for metabolic power analysis (Osgnach et al. [3])	Correlations between metabolic power categories and aerobic fitness variables were: large for VO_2max_ (*r* = 0.55–0.68) and %VO_2_VT (*r* = 0.62–0.65), very large for VO_2_VT (*r* = 0.72–0.83), and large to very large for maximal aerobic speed (*r* = 0.52–0.72) and V_L4_ (*r* = 0.56–0.73)
Oxendale et al. [33]	12 university team sport players (rugby, soccer, hockey, netball; males, 5 females; 20.8 ± 2.7 years)	3 different testing protocols: (1) 20 m shuttle fitness test with progressively increasing speed to determine VO_2max_; (2 & 3) multi-directional and linear circuit including 8 bouts of 60 s of intermittent activities (running, sprinting) followed by 120 s rest	GPS (4 Hz) derived EE compared to VO_2_ (portable metabolic cart) derived EE; use of modified equation for metabolic power analysis (Osgnach et al. [3])	GPS derived EE was lower during multi-directional (−52%) and linear (−34%) condition; GPS and VO_2_ derived EE was strongly correlated for multi-directional (*r* = 0.89) and linear (*r* = 0.95) condition
Stevens et al. [34]	14 amateur soccer players of unknown sex (23 ± 2 years)	2 sessions separated by 30 min on artificial turf: (1) 10 m continuous shuttle running and constant running following the same protocol of running with increasing speed (0.5 km/h every 3 min) from 7.5 to 10 km/h; (2) incremental protocol with an increase of 1 km/h per min until exhaustion followed immediately after the constant running	LPS (500 Hz filtered at 10 Hz) derived EC compared to VO_2_ (portable metabolic cart) derived EC; determining additional energy cost of 180° change of direction compared to constant running; use of original equation for metabolic power analysis (di Prampero et al. [15]) with adaptations concerning EC of running on flat terrain at constant speed and KT	LPS derived EC was significantly higher (6–11%, main effect: 0.34, *p* < 0.001) in constant running and lower (−13 to −16%, main effect: −0.94, *p* < 0.001) in shuttle running when compared to VO_2_ derived EC

*CV*  coefficient of variation, *EC*  energy cost, *EE*  energy expenditure, *GPS*  global positioning system, *Hz*  hertz, *ICC*  intraclass coefficient correlation, *iTRIMP*  individualized training impulse, *km/h*  kilometers per hour, *KT*  terrain constant, *LPS*  local positioning system, *m*  meters, min  minutes, *mmol/l*  millimoles per liter, *s*  seconds, *V*_*L4*_  running speed at blood lactate concentration of 4 mmol/l, *VO*_*2*_  oxygen uptake, *VO*_*2max*_  maximum oxygen uptake, *VO*_*2*_*VT*  oxygen uptake at ventilatory threshold, *%VO*_*2*_*VT*  percentage of oxygen uptake at ventilatory threshold, *W/kg*  watts per kilogram

Table 4 presents the 13 methodological validation studies. Soccer was the primary investigated sport including 10 studies [35, 36, 38–45] followed by rugby with 2 studies [37, 47] and hockey with 1 study [46]. Concerning sex, 10 studies did not specify [36–45], whereas 3 studies investigated males [35, 46, 47]. Adults were examined in 8 studies [35, 37–39, 42, 45–47] and 5 studies were based on youth players [36, 40, 41, 43, 44]. Regarding the playing level, professional players were investigated by 10 studies [35–39, 41, 42, 45–47]. Two studies addressed sub-elite players [43, 44], while 1 study did not specify the playing level [40].

**Table 4:** Characteristics and synthesis of the methodological validation studies based on the PICO scheme

Study (Year)	Population	Intervention	Comparison	Outcome
Castagna et al. [35]	1200 male first division German, English, and Spanish soccer players (24.5 ± 0.8 years)	Data from 20 (out of 60) randomly selected official matches	Comparison of metabolic power and traditional running speed approach using a video camera system (25 Hz); parameters were distance covered at HI (≥ 16.0 km/h), HIR (≥ 18.0 ≤ 22.0 km/h), VHIR (≥ 22.0 km/h), HIAcc (≥ + 2 m/s^2^), VHIAcc (≥ + 3 m/s^2^), HIDec (≤ −2 m/s^2^), VHIDec (≤ −3 m/s^2^), MPHI (≥ 20.0 W/kg) as well as TD and AMP; use of modified equation for metabolic power analysis (Osgnach et al. [3])	High inter-match variations (CV > 10%) for all parameters except TD, AMP, and MPHI; significant measurement bias (effect size = 11.67) between MPHI and HI distances; nearly perfect correlation (*r* = 0.93) between MPHI and HI; very large correlations between MPHI and TD (*r* = 0.84), HIDec (*r* = 0.73), and HIR (*r* = 0.87) as well as between AMP and HI (*r* = 0.73), TD (*r* = 0.85), VHIDec (*r* = 0.72), and HIDec (*r* = 0.76)
Darbellay et al. [36]	14 elite youth Swiss soccer players of unknown sex (17 ± 1 years)	Data from 13 official matches and 2 SSGs	Comparison of metabolic power and traditional running speed approach (fixed and individual speed zones) using a GPS (10 Hz); e.g., high intensity zone: 20.0–35.0 W/kg vs. 16.0–19.0 km/h; use of modified equation for metabolic power analysis (Osgnach et al. [3])	Matches: significantly higher distance covered in intermediate and high-intensity zones for metabolic power compared to running speed methods (*p* ≤ 0.001) and significantly higher (individual speed) and lower (fixed speed) for very high-intensity zones (*p* ≤ 0.001); SSG: significantly higher distance in high and very high-intensity zones for metabolic power compared to speed methods (*p* ≤ 0.002)
Dubois et al. [37]	14 professional French rugby union players of unknown sex (24.1 ± 3.4 years)	Data from 5 official matches during European Challenge Cup	Comparison of metabolic power, traditional running speed, and heart-rate-based approach using a GPS (5 Hz interpolated to 15 Hz) and heart rate monitors; thresholds: > 20.0 W/kg, > 14.4 km/h, and 85% of HR_max_; use of modified equation for metabolic power analysis (Osgnach et al. [3])	Near perfect correlation between total distance (traditional) and estimated distance (metabolic power) (*r* = 0.98) and between high-speed running and high metabolic power distance (*r* = 0.93); percentage differences between traditional and metabolic power approach during high-intensity running (up to + 53%)
Gaudino et al. [38]	26 professional English soccer players of unknown sex (26 ± 5 years)	Data from 3 different SSGs (5 vs. 5, 7 vs. 7, 10vs10) (420 individual observations; median of 16 drills per player)	Comparison of metabolic power and traditional running speed approach using a GPS (15 Hz); thresholds: > 20.0 W/kg and > 14.4 km/; use of modified equation for metabolic power analysis (Osgnach et al. [3])	Distance at high-metabolic power was significantly higher compared to high-speed running regardless of SSGs (99%, *p* < 0.001; effect size = 0.8); percentage of high-metabolic power was higher compared to high-speed running during all SSGs (*p* < 0.001; effect size = 1.9–2.8); differences decreased from 5vs5 to 10vs10 (*p* < 0.01; effect size = 0.6–1.0)
Gaudino et al. [39]	26 professional English soccer players of unknown sex (26 ± 5 years)	Data from a 10-week training period (638 individual observations; median of 24 training sessions per player)	Comparison of metabolic power and traditional running speed approach using a GPS (15 Hz); thresholds: > 20.0 W/kg and > 14.4 km/h as well as 3 different high-speed and high-metabolic power categories; use of modified equation for metabolic power analysis (Osgnach et al. [3])	Distance at total high-metabolic power was significantly higher compared to total high-speed running (*p* < 0.001; effect size = 0.8); relation between both methods decreased as high-intensity distance increased (*R*^2^ = 0.43; *p* < 0.001)
Goto and King [40]	11 youth soccer players of unknown sex (16 ± 0.6 years)	3 different pitch sized SSGs (975, 1980, 3900 m^2^) and a match each lasting 35 min; conducted 4 times during 6 weeks	Examination of difference between metabolic power and traditional running speed approach using a GPS (5 Hz interpolated to 15 Hz); thresholds: ≥ 20.0 W/kg and ≥ 15.5; use of modified equation for metabolic power analysis (Osgnach et al. [3])	Distance at high-metabolic power was significantly higher compared to high-speed running in all SSG and match (*p* < 0.001; effect size = 1.3–1.9); differences decreased with increase in pitch size during SSGs (615–102%), difference in match was 145%
Goto and Saward [41]	110 professional youth Japanese soccer players from U13–U18 of unknown sex (12.2–18.7 years)	Data from 48 official league matches	Examination of age-related differences in running performance; comparison of metabolic power and traditional running speed approach using a GPS (5 Hz interpolated to 15 Hz); thresholds: ≥ 20.0 W/kg and ≥ 14.4 km/h; use of modified equation for metabolic power analysis (Osgnach et al. [3])	Distance at high-metabolic power was significantly higher compared to high-speed running in all age-groups (*p* < 0.01; effect size = 0.49–0.61); percentage difference decreased with increasing age (*p* < 0.001; effect size = 0.63); moderate negative correlation between percentage difference and age (*p* < 0.001; *r* = −0.45)
Hoppe et al. [42]	12 professional German soccer players of unknown sex (26 ± 3 years)	Data from 5 pre-season matches; only data of completed halves were analyzed (total of 61 halves)	Examination of intraindividual variability of metabolic power using a GPS (10 Hz); comparison of variability of high metabolic power (≥ 20.0 W/kg), speed (≥ 15.5 km/h), acceleration (≥ + 3 m/s^2^), and deceleration (≤ −3 m/s^2^); use of modified equation for metabolic power analysis (Osgnach et al. [3])	Variability of global metabolic power data (EE, EC, AMP; CV = 0.8–11.4%) was lower than high-intensity (high and peak metabolic power; CV = 6.1–50.0%); variability of high metabolic power (CV = 14.1 ± 3.5%) was comparable to high speed (17.0 ± 6.2%), acceleration (11.1 ± 5.1%), and deceleration (11.9 ± 4.5%)
Lord et al. [43]	20 sub-elite youth soccer players of unknown sex (19.1 ± 1.2 years)	4 competitive matches and 3 field-based test sessions; (1) maximal straight-line running efforts: 400 m running track and efforts over 40, 100, and 400 m; (2) critical speed field test—straight line: 400 m running track and efforts over 1200, 2400, and 3600 m continuous running; (3) critical speed field test—shuttle running: 100 m straight line track and maximal shuttle-runs over 100, 400, and 1500 m	Examination of validity and reliability of maximal speed, maximal metabolic power, critical speed, and critical metabolic power using a GPS (15 Hz); differences therein during matches versus field-based maximal effort running tests; use of original equation for metabolic power analysis (di Prampero et al. [15])	Validity: critical speed and critical metabolic power showed a good correlation (*r* = 0.843); critical speed (*p* = 0.066) and critical metabolic power (*p* = 0.271) showed no difference to shuttle-run data; Reliability (match): ICC was large (0.577) to nearly perfect (0.902) for speed and very large (0.701–0.863) for metabolic power data; CV was moderate to good for speed (3.8–5.6%) as well as metabolic power (3.9–7.8%) data
Lord et al. [44]	20 sub-elite youth soccer players of unknown sex (19.1 ± 1.2 years)	Data from 26 official matches (416 individual match samples)	Examination of match-to-match variations of match running performance (distances, maximal maintainable speed, and metabolic power) over 2–10 matches using a GPS (15 Hz); use of original equation for metabolic power analysis (di Prampero et al. [15])	Match-to-match variations for maximal speed (CV = 4.9–7.0%) and maximal metabolic power (CV = 4.4–9.6%) were good to moderate
Martinez-Cabrera and Núnez-Sánchez [45]	38 professional Romanian soccer players of unknown sex (26.3 ± 3.9 years)	Data from 18 pre-season matches (over 4 years); total of 300 individual observations; only data of completed halves were analyzed; grouped according to playing position	Comparison of metabolic power and traditional running speed approach (fixed speed/metabolic power zones) using a GPS (15 Hz); e.g., 20.1–35.0 W/kg vs. 16.1–19.0 km/h; use of original equation for metabolic power analysis (di Prampero et al. [15])	No differences were found between metabolic power and traditional running speed approach concerning high, medium, and low intensities in different playing positions using absolute values only
Polglaze et al. [46]	12 male elite Australian hockey players (25.5 ± 4.5 years) Two-part study, only 10 of 12 participants in part 2	Two field tests (series of time trials, 3 min all-out shuttle running) Data from two international hockey matches	Comparison of critical metabolic power and critical speed using a GPS (10 Hz) as well as time above 85% HR_max_; use of original equation for metabolic power analysis (di Prampero et al. [15])	Correlation for critical metabolic power was very large in the two field tests (*r* = 0.754; *p* = 0.005); in matches, the correlation between time above 85% HR_max_ and critical metabolic power was very large (*r* = 0.867, *p* < 0.001)
Scott et al. [47]	26 male professional American rugby league players (26.4 ± 3.7 years)	Data from 25 official matches (346 individual observations); 30–15 intermittent fitness test four times during season to identify first and second ventilatory threshold as well as high metabolic power threshold; grouped according to playing position	Comparison of metabolic power approach and relative and absolute speed using a GPS (5 Hz interpolated to 15 Hz); thresholds: > 20.0 W/kg, 13.0 km/h (MIR), and 18.7 km/h (HIR); use of modified equation for metabolic power analysis (Osgnach et al. [3])	Strong positive relationship between absolute and relative measures: V_1IFT_ and MIR (*r* = 0.94), V_2IFT_ and HIR (*r* = 0.94), and HP_metVT2_ and HP_metTh_ (*r* = 0.93); HP_metVT2_ was likely to almost certainly be lower than HP_metTh_ in all playing positions (effect size = 0.24–0.63); absolute MIR and high metabolic thresholds may over- or underestimate the load depending on the respective fitness of the individual

*AMP*  average metabolic power, *CV*  coefficient of variation, *EC*  energy cost, *m*  meters, *EE*  energy expenditure, *GPS*  global positioning system, *HI*  high intensity, *HIAcc*  high intensity acceleration, *HIDec*  high intensity deceleration, *HIR*  high intensity running, *HP*_*metTh*_  absolute high metabolic power threshold, *HP*_*metVT2*_  relative high metabolic power threshold (power associated with VT_2IFT_), *HR*_*max*_  maximum heart rate, *Hz*  hertz, *ICC*  intra-class correlation, *km/h*  kilometers per hour, *m*^*2*^  square meters, *min*  minutes, *MIR*  moderate intensity running, *MPHI*  high intensity metabolic power, *m/s*^*2*^  meters per second squared, *R*^*2*^  regression coefficient, *SSG*  small sided game, *TD*  total distance, *V*_*1IFT*_  first ventilatory threshold based on the 30–15 intermittent fitness test, *V*_*2IFT*_  second ventilatory threshold based on the 30–15 intermittent fitness test, *VHIAcc*  very high intensity acceleration, *VHIDec*  very high intensity deceleration, *VHIR*  very high intensity running, *W/kg*  watts per kilogram

Table 5 shows the characteristics of the 11 conceptual studies. Three studies were based on theoretical analyses and thus did not include any subjects [16, 17, 19]. Two further studies included data sets from other studies, of which 1 study focused on track [8] and the other on soccer [50] with no detailed subject descriptions. Of the remaining 6 studies, soccer was the most investigated sport including 5 studies [3, 48, 49, 52, 53] followed by 1 study on tennis [51]. Regarding sex, males and females were examined in 3 [48, 51, 52] and 2 studies [49, 53], respectively. Sex was not specified in 1 study [3]. Adults were studied in 4 studies [3, 48, 49, 52], while 1 study was on youth players [51]. The age of the subjects was not stated in 1 study [53]. All of these studies examined professional players.

**Table 5:** Characteristics and synthesis of the conceptual studies based on the PICO scheme

Study (Year)	Population	Intervention	Comparison	Outcome
Di Prampero et al. [8]	12 medium-level sprinters (from previous study by di Prampero et al. [15]) and data for Usain Bolt	Summary of theoretical aspects underlying the metabolic power approach	Practical conclusions such as implementation in GPS software, estimation of actual VO_2_, comparison of actual and estimated VO_2_, comparison of mechanical accelerating power of medium-level sprinters and soccer players to Usain Bolt; use of original equation for metabolic power analysis (di Prampero et al. [15])	GPS (20 Hz) derived, actual VO_2_ consumed was close to VO_2_ determined by portable metabolic carts
Di Prampero and Osgnach [17]	/	Theoretical assumptions to update metabolic power approach	Extension of metabolic power approach by addressing (1) air resistance and (2) differences between running and walking periods	Air resistance: equation for calculating ES was extended by addition of a second equivalent slope equation: ES_D_ = k*v^2^*g^−1^, effects of air resistance are minor and only amount of ~ 2% of total energy expenditure; Walking periods: new equation when locomotion is identified as walking: ECw_vES_ = (ECw_vLES_ + ΔECw_v_*(ES-LES)*(HES-LES)^−1^)*EM, effects on whole match energy expenditure are ~ 14% smaller than previously obtained
Gaudino et al. [48]	29 professional male soccer players (19 ± 1 years)	Maximum sprint (12 m) and shuttle test with 180° change of direction (12 + 12 m) on different terrains (grass, artificial turf, sand)	Energetic and biomechanical variations in sprints with and without change of direction on different terrains using a GPS (5 Hz); use of modified equation for metabolic power analysis (Osgnach et al. [3])	Modified equation was extended by multiplication of an additional constant (KT = 1.45) for calculating EC on sand; EC and metabolic power were highest, while speed and acceleration were lowest on sand (*p* < 0.001); no significant differences between grass and artificial turf (*p* > 0.5)
Gray et al. [16]	/	Theoretical alternative energetic approach	Attempt to further quantify energetic costs of team sports especially during collisions via a mechanical modeling approach	Metabolic power approach shows limitations especially in collisions-based sports; alternative approach to derive energetic demands through mechanical work (external work + internal work) which can be predicted by obtaining speed and/or acceleration data
López-Fernández et al. [49]	16 Spanish 2nd Division female soccer players (20 ± 2 years)	SSG on different terrains (ground, grass, artificial turf) and different pitch sizes (400, 600, 800 m^2^)	Metabolic power demands of SSG played on different terrains using a GPS (5 Hz interpolated to 15 Hz); use of modified equation for metabolic power analysis (Osgnach et al. [3])	All metabolic variables were significantly lower (*p* < 0.05) on ground compared to all pitch sizes on grass and all except smallest pitch size on artificial turf
Osgnach et al. [3]	399 Italian elite soccer players of unknown sex (27 ± 4 years)	Data from 56 competitive matches during one season	Match performance based on speed, acceleration, and metabolic power using a video camera system (25 Hz); EC and metabolic power were calculated via metabolic power approach; use of original equation for metabolic power analysis (di Prampero et al. [15])	Original equation was extended by multiplication of a constant (KT = 1.29) to take different terrain into account (grass vs. treadmill); mean EE during match play is 14.60 ± 1.57 kcal/kg
Osgnach and di Prampero [50]	Subjects from 2, then unpublished, studies: 1. soccer players (no further description); 2.497 Italian outfield soccer players	Theoretical approach as well as data from then unpublished studies	Estimation of corresponding time course of actual VO_2_, aerobic and anaerobic energy supply as well as high and low intensity energy bouts	Equation for estimating actual VO_2_ kinetics: VO_2_T_n(t)_ = (E_n_−VO_2_T_n(0)_)*(1−e^−t/T^) + VO_2_T_n(0)_; anaerobic or aerobic energy supply is given when metabolic power requirement is greater or lower than actual VO_2_; 5-step procedure to identify high and low intensity energy bouts (excess of a defined threshold, duration, peak and mean power, subsequent bouts, low intensity)
Polglaze and Hoppe [19]	/	Summary of metabolic power approach and its limitations and benefits as well as future perspectives	/	Metabolic power approach addresses energetic cost of changing speed by analyzing interaction between speed and acceleration but it is not capable to estimate overall energy expenditure or mechanical work; distinction between acceleration conducted at different starting speeds; validity of metabolic power approach: VO_2_ and metabolic power cannot simply be compared as VO_2_ shows aerobic, whereas metabolic power shows aerobic and anaerobic contribution; metabolic power is a sensible tool to quantify intensity in team sports with the potential to use individualized thresholds
Ponzano and Gollin [51]	12 nationally ranked male tennis players (16 ± 3 years)	Data from 24 matches with 12 matches being played on red clay and on hard court each	Analysis of speed, heart rate, acceleration, deceleration, metabolic power using a GPS (15 Hz); use of original equation for metabolic power analysis (di Prampero et al. [15])	Mean metabolic power was significantly higher (*p* < 0.05, effect size = 0.72) on clay (3.9 ± 0.3 W/kg) compared to hard court (3.7 ± 0.3 W/kg)
Savoia et al. [52]	Two-part study: (1) 17 Italian professional male soccer players (24 ± 3 years), (2) 13 out of the 17 players of first part of study (22 ± 6 years)	Assessment of VO_2max_ on a treadmill run to exhaustion; (1) 6 min aerobic-based steady-state run at 10.29 km/h on a 160 m circular course; (2) 8 min soccer-specific run at varying speeds	Determination of energy cost of running on grass as well as updating and validating the metabolic power equation using a GPS (10 Hz)	Energy cost of running on grass was 4.7 J/kg/m; converting metabolic power algorithm to a new on including energy cost of running on grass: EC = 30.4x^4^−5.0975x^3^ + 46.3x^2^ + 17.696 + 4.66; correlation between metabolic power via VO_2_ and via GPS with new equation as well as via GPS with old equation was 0.66 and 0.63, respectively; estimates of fixed and proportion bias were negligible in both approaches; significant difference between metabolic power via VO_2_ and via GPS with old equation (*p* < 0.001) and no difference between metabolic power via VO_2_ and via GPS with new equation (*p* = 0.853)
Vescovi and Falenchuk [53]	28 professional female soccer players of unknown age	Data from official matches	Examination of impact of different surfaces (natural vs. artificial turf) on metabolic power distances using a GPS (5 Hz); use of modified equation for metabolic power analysis (Osgnach et al. [3])	High-, elevated-, and maximal-metabolic power distances were elevated on artificial turf compared to natural turf (*p* = 0.004, 0.097, 0.239, respectively)

*EC*  energy cost, *ECw*_*vES*_  energy cost of walking at the actual speed and ES, *ECw*_*vLES*_  energy cost of walking at the actual speed and lower ES, *ΔECw*_*v*_  difference between ECw corresponding to the closest higher and lower ES functions, *EE*  energy expenditure, *EM*  equivalent mass, *ES*  equivalent slope, *ES*_*D*_  equivalent slope with air drag, *GPS*  global positioning system, *HES*  higher equivalent slope, *Hz*  hertz, *J/kg/m*  Joules per kilogram per meter, *kcal/kg*  calories per kilogram, *km/h*  kilometers per hour, *KT*  terrain constant, *LES*  lower equivalent slope, *m*  meters, *min*  minutes, *SSG*  small-sided games, *VO*_*2*_  oxygen uptake, *VO*_*2max*_  maximum oxygen uptake, *VO*_*2*_*T*_*n(0)*_  theoretical VO_2_ value at onset of metabolic power interval, *VO*_*2*_*T*_*n(t)*_  theoretical VO_2_ value at time t of a metabolic power interval, *W/kg*  watts per kilogram

### Synthesis of Physiological Validation Studies

Table 3 summarizes the 7 physiological validation studies according to the PICO scheme.

Of these 7 studies, the gold standard for validating tracking-based metabolic power was the use of oxygen uptake determined by portable metabolic carts in 6 studies [29–34], while lactate parameters were applied in 1 study [28]. As tracking technologies, 4–15 Hz GPS [28–31, 33], 10 Hz LPS devices [34], and a 25 Hz video camera system [32] were used in 5, 1, and 1 studies, respectively.

Concerning the interventions, 4 studies used constant or shuttle running test protocols until exhaustion [28, 32–34]. Specific sports circuits were utilized in 5 studies [28–31, 33], while 1 study applied official match play data [32].

Comparison between energy expenditure measured by oxygen uptake and GPS derived metabolic power during sport circuits showed lower results for the metabolic power approach in 4 studies [29–31, 33]. Conversely, during walking, energy expenditure was higher when measured with the metabolic power approach via GPS compared to oxygen uptake as revealed in 1 study [29]. The energy cost by LPS derived metabolic power was higher in constant and lower in shuttle running compared to energy cost via oxygen uptake as shown in 1 study [34]. Concerning the correlation between GPS or video camera system derived metabolic power and oxygen uptake, moderate [30, 31] and large [32, 33] relations were found in 2 studies, respectively. Moreover, 1 study showed small to large relationships between GPS derived metabolic power and lactate parameters [28].

### Synthesis of Methodological Validation Studies

Table 4 shows the summary of the 13 methodological validation studies based on the PICO scheme.

The most frequently used gold standard for validating the tracking-based metabolic power was the traditional running speed analysis including 8 studies [35–41, 45]. Further gold standards were the critical speed approach with 2 studies [43, 46] and the use of relative and absolute running speed based on first and second ventilatory thresholds with 1 study [47]. Furthermore, the metabolic power approach was examined regarding intraindividual [42] and match-to-match variability [44] in 1 study each. As tracking technologies, 10–15 Hz GPS devices [36–47] and a 25 Hz video camera system [35] were used in 12 and 1 studies, respectively.

Regarding the interventions, official matches were investigated in 10 studies [35–37, 41–47] and small-sided games were inspected in 3 studies [36, 38, 40]. Shuttle running efforts were tested in 2 studies [43, 46]. Training sessions [39], modified matches [40], straight-line running efforts [43], intermittent fitness tests [47], and time trials [46] were applied in 1 study each.

Concerning the validation of the metabolic power approach to the traditional running speed approach, 6 studies showed that the distance covered in high-intensity zones was significantly higher for GPS derived metabolic power than for running speed [36–41]. One study found no differences between the two approaches in different intensity zones regarding playing positions [45]. Furthermore, 1 study showed that relative high metabolic threshold was likely to almost certainly be lower than absolute metabolic threshold during rugby matches [47]. A nearly perfect correlation between high intensity running and GPS or video camera system derived high intensity metabolic power distances was found in 2 studies [35, 37]. Comparison to the critical speed approach revealed a very large correlation between critical speed and GPS derived critical metabolic power [46] and a very large correlation of GPS derived critical metabolic power between different field tests [43] in 1 study each. Two further studies discovered a moderate correlation of GPS derived metabolic power between different matches [42, 44].

### Synthesis of Conceptual Studies

Table 5 shows the summary of the 11 conceptual studies based on the PICO scheme.

Three studies addressed the metabolic power approach by extending the original equation based on air resistance [17], grass [3], and sand [48]. Additionally, the first study presented a solution to differentiate between running and walking periods [17]. Another study developed a new equation to estimate metabolic power following the original concept of the metabolic power approach [52]. The effects of different terrains were investigated in 5 studies [3, 48, 49, 51, 53], while collisions were examined in 1 study [16]. Furthermore, 1 study focused on the identification of high and low intensity energy bouts [50]. Finally, practical conclusions for the application of the metabolic power approach were given in 2 studies [8, 19].

The studies executing practical approaches included match data with 3 studies [3, 51, 53] as well as sprint and shuttle tests [48], small-sided games [49], treadmill tests to exhaustion [52], aerobic steady-state runs [52], and soccer-specific runs [52] with 1 study each using GPS (5–15 Hz) or a video camera system (25 Hz).

The investigation of 1 study on the effects of air resistance on metabolic power showed that these effects were negligible [17]. However, the same study stated that with the inclusion of walking periods, the former energy expenditure was overestimated. The examination of different terrains, as conducted in 2 studies, showed that running on grass is 1.29 [3] and on sand an additional 1.45 [48] times more energetically demanding compared to running on a treadmill. One study converted the original equation into a new equation, which both showed similar moderate correlations with oxygen uptake. However, there was no significant difference between the new equation and oxygen uptake, while a significant difference was found between the old equation and oxygen uptake [52]. Concerning the influence of different terrains, GPS derived metabolic power was significantly higher on sand compared to grass and artificial turf [48] and on clay compared to hard court [51] as well as significantly lower on ground compared to grass and artificial turf [49] as shown by 1 study, respectively. Another study showed that GPS derived metabolic power distances were higher on artificial than natural turf [53]. To include the impact of collisions, 1 study applied a mechanical work approach combining external and internal workloads based on speed and/or acceleration data [16]. Moreover, 1 study established an equation on the kinetics of oxygen uptake to detect the phases of aerobic and anaerobic energy supply and thereafter, following a 5-step procedure to differentiate between high and low intensity energy bouts [50]. Regarding the practical conclusions of the metabolic power approach, 1 study showed that GPS (20 Hz) derived oxygen uptake was similar to that through portable metabolic carts [8]. However, another study indicated that oxygen uptake and metabolic power cannot simply be compared because oxygen uptake only represents aerobic, while metabolic power represents both aerobic and anaerobic contributions [19]. Finally, the same study emphasized that the metabolic power approach is incapable of estimating overall energy expenditure or mechanical workload.

### Synthesis of Results

Tables 6 and 7 show the results of the evidence assessment of the physiological and methodological validation studies, respectively.

**Table 6:** Results of the physiological validation studies using a best-evidence synthesis

Study (Year)	Criterion	Association	Study quality	Rating
*Comparison of metabolic power with (validity)*				
Brown et al. [29]	Oxygen uptake	Lower via GPS derived metabolic power	High	Strong evidence
Buchheit et al. [30]		Lower via GPS derived metabolic power	High	
Highton et al. [31]		Lower via GPS derived metabolic power	High	
Oxendale et al. [33]		Lower via GPS derived metabolic power	High	
Stevens et al. [34]		Lower via LPS derived metabolic power	High	
*Correlation of metabolic power with (validity)*				
Buchheit et al. [30]	Oxygen uptake	Small to moderate	High	Conflicting evidence
Highton et al. [31]		Moderate	High	
Manzi et al. [32]		Large	High	
Oxendale et al. [33]		Large	High	
Akubat et al. [28]	Lactate parameters	Moderate to large	High	Moderate evidence

Study quality was assessed using the Downs and Black checklist

**Table 7:** Results of the methodological validation studies using a best-evidence synthesis

Study (Year)	Criterion	Association	Study quality	Rating
*Comparison of metabolic power with (validity)*				
Darbellay et al. [36]	High-speed running	Higher via GPS derived metabolic power	High	Strong evidence
Dubois et al. [37]		Higher via GPS derived metabolic power	High	
Gaudino et al. [38]		Higher via GPS derived metabolic power	Moderate	
Gaudino et al. [39]		Higher via GPS derived metabolic power	Moderate	
Goto and King [40]		Higher via GPS derived metabolic power	Moderate	
Goto and Saward [41]		Higher via GPS derived metabolic power	High	
Martinez-Gabrera and Núnez-Sánchez [45]		No differences	High	
*Correlation of metabolic power with (validity)*				
Castagna et al. [35]	High-speed running	Very large – near perfect	High	Strong evidence
Dubois et al. [37]		Near perfect	High	
Polglaze et al. [46]	Critical speed	Very large	High	Moderate evidence
Scott et al. [47]	Relative and absolute speed	Strong	High	Moderate evidence
*Correlation of metabolic power within (reliability)*				
Lord et al. [43]	Critical metabolic power	Very large	High	Moderate evidence
Hoppe et al. [42]	Metabolic power	Moderate	High	Strong evidence
Lord et al. [44]		Good to moderate	High	

Study quality was assessed using the Downs and Black checklist

Concerning the physiological validity, there was strong evidence that energy expenditure is lower when determined via GPS or LPS derived metabolic power compared to oxygen uptake by portable metabolic carts during intermittent running activities [29–31, 33, 34]. The correlation of metabolic power with oxygen uptake showed conflicting evidence as it ranges from small to large [30–33]. Additionally, the correlation with lactate parameters, which are moderate to large, disclosed moderate evidence [28].

In terms of the methodological validity, strong evidence was shown that energy expenditure via GPS derived metabolic power is higher than that via high-speed running [36–41, 45]. Similarly, the correlation with high-speed running presented strong evidence as it is very large to near perfect [35, 37]. Moderate evidence was revealed in terms of the correlation of critical metabolic power with critical speed [46] as well as with relative and absolute running speed [47]. There was moderate evidence concerning the correlation of the metabolic power within critical metabolic power [43]. Finally, the correlation within metabolic power, being moderate, showed strong evidence [42, 44].

## Discussion

To the best of our knowledge, this is the first systematic review aimed to present the validity and evidence of the metabolic power approach that was first introduced by di Prampero et al. [15] in 2005 for estimating metabolic loads in intermittent team and racquet sports. Based on the best-evidence synthesis, the main findings were that (1) conflicting to strong evidence was shown concerning the physiological validity and (2) moderate to strong evidence was revealed regarding the methodological validity. Additionally, the conceptual studies showed that (3) the distinction between walking and running episodes, different terrains, as well as aerobic and anaerobic energy supply should be considered when analyzing metabolic power in intermittent sports.

Concerning the characteristics of the 7 physiological validation, 13 methodological validation, and 11 conceptual studies, the most investigated sport was soccer. The subjects were predominantly male adult professional players. For validation studies, the gold standards most commonly used for tracking-based metabolic power were oxygen uptake via portable metabolic carts and traditional running speed analysis. The tracking technology predominately used in these studies was GPS operating between 4 and 15 Hz. While physiological validation mainly focused on sport specific tests, match data were primarily examined in methodological validation studies. Updating the original concept was mostly based on the distinction between running and walking episodes, different terrains as well as aerobic and anaerobic energy supply (Tables 3, 4, and 5). These characteristics show a lack of research concerning children, females, and intermittent sports besides soccer and the application of more profound physiological approaches for the validation and assessment of tracking technology-based metabolic power is needed. This should be considered when planning and conducting future studies.

The first main finding of this study was that the physiological validity of the metabolic power approach showed conflicting to strong evidence (Table 6). However, all studies were high quality (Table 2). Strong evidence was shown that supposedly GPS or LPS derived metabolic power underestimates energy expenditure during team sport specific test protocols compared to that derived via oxygen uptake, especially concerning multidirectional activities [29–31, 33, 34]. In contrast, Brown et al. [29] disclosed an overestimation of GPS derived energy expenditure via metabolic power during walking episodes. However, the original and modified approaches by di Prampero et al. [15] and Osgnach et al. [3], respectively, were simply incapable of distinguishing between walking and running episodes. Regarding the update in 2018 [17], this limitation has been solved. Furthermore, certain studies tried validating activities that could not be registered by tracking devices (e.g., collisions, running with the ball, running backward or sideways) [30, 31]. Therefore, the resulting lower energy expenditure of GPS or LPS derived metabolic power is reasonable. When considering the correlation between the metabolic power approach and oxygen uptake, conflicting evidence was shown varying from small to large correlations. This indicates the inconsistency of the results regarding the validation of the metabolic power approach against oxygen uptake, especially considering that, in intermittent running, oxygen uptake only discloses aerobic energy supply, whereas metabolic power contains both aerobic and anaerobic energy supply [19]. Additionally, the inclusion of energy expenditure during passive resting periods when comparing oxygen uptake to the metabolic power approach is inappropriate [50, 54]. As the estimation of energy expenditure via tracking-based metabolic power is based on acceleration and speed, no data can be received during passive resting periods, where oxygen uptake is still elevated to balance the oxygen uptake deficit [50]. A further physiological validation study included the use of lactate parameters [28]. The correlation with metabolic power was moderate to large showing moderate evidence. However, as there was only one study including lactate parameters, oxygen uptake is still most often used when physiologically validating the metabolic power approach, regardless of its limitations when used during intermittent running. Overall, the studies showed that previous physiological validations are outdated as there have been adaptations concerning the metabolic power approach. Additionally, the implementation of the validation was often inappropriate due to the inclusion of, e.g., collisions or resting periods.

The second main finding was that moderate to strong evidence was revealed regarding the methodological validation of the metabolic power approach (Table 7). Three studies were of moderate quality, whereas the remaining studies were of high quality (Table 2). Strong evidence was shown that distances covered at high metabolic power are greater compared to high-speed running during matches or training. However, there were inconsistencies regarding the calculated threshold of high-speed running when comparing with an energetic equivalent metabolic power threshold. While the actual equivalent to 20 W/kg for constant speed running is 15.5 km/h [19], most studies used 14.4 km/h as a threshold [37–39, 41]. Consequently, the discrepancy between the two approaches regarding distances covered above these thresholds is likely greater than reported. This indicates that the identification of high-speed running alone does not show the full extent of the contribution to energy expenditure or rather the intensity of matches or training. Indeed, activities such as accelerations during lower speed largely contribute to high metabolic power and are important to monitor [19]. This shows the advantage of the metabolic power over the traditional running speed approach. Additionally, the correlation between high-speed running and high metabolic power was reported as very large to near perfect [35, 37] and thus showed strong evidence. This result was also shown in the correlation between critical speed and critical metabolic power as the relationship was very large [46]. Because there was only one study utilizing these parameters, the evidence was moderate. Concerning the reliability of the metabolic power approach, there was moderate evidence that the approach is very largely correlated within critical metabolic power [43] and good to moderately correlated within metabolic power itself [42, 44], which is a prerequisite for the validity of the approach. Collectively, there is strong evidence that the metabolic power approach is valid from this perspective and, regarding the traditional running speed approach, a superior method to monitor metabolic loads during matches and training in intermittent sports.

The last main finding was that the differences between walking and running episodes, different terrains, as well as between aerobic and anaerobic energy supply should be noted in terms of validation purposes as shown by the conceptual studies (Table 5). As revealed by a physiological validation study discussed above [29], the original metabolic power approach seemed to overestimate energy expenditure in terms of walking episodes compared to energy expenditure via oxygen uptake. However, contrary to walking, in running due to the flight phase between steps, part of the kinetic energy from each step is absorbed by active muscles and tendons and retained as mechanical energy for the next step [55]. Hence, the relative energy cost of running but not walking is independent of speed [18]. Therefore, an additional equation was established to account for walking episodes [17]. Another factor addressed in the conceptual studies is the influence of the underlying terrain. Depending on the surface, the estimated energy expenditure differs [48, 49, 51, 53]. To increase comparability between studies on different terrains, the original metabolic power equation was extended by individual factors regarding the different surfaces. However, only specific correction factors for grass and sand have been developed to date [3, 48]. In this context, the consideration of real surface adjustments based on Clegg hammer measurements to obtain more precision about surface rigidity is logical [56]. Lastly, an adaptation of the metabolic power approach was conducted regarding the separation of aerobic and anaerobic energy supply [50]. When maximum oxygen uptake is known, net oxygen uptake can be simulated as there is a known time delay of approximately 20 s between the oxygen uptake kinetics at the muscle and upper airway. Then, the metabolic origin can be distinguished as either aerobic or anaerobic when metabolic power is lower or higher than the simulated actual oxygen uptake, respectively [50]. In intermittent sports, different ways of energy supply are implicated [57, 58]. Therefore, the knowledge of energy expenditure derived from aerobic or anaerobic supply can provide a more relevant overview of the metabolic load. In addition to these conceptual aspects, the validity of the tracking technology as well as the impact of the sampling rate and filtering techniques to assess and process the required acceleration data should be considered when discussing the validity of the metabolic power approach. In fact, an important prerequisite is to assess valid acceleration data and reduce the noise without losing information for which no established procedures exist as yet [42, 54].

Overall, the metabolic power approach has recently evolved. In particular, the differentiation between walking and running episodes needs to be considered when using the metabolic power approach to, e.g., track matches where characteristically, walking episodes are present between running bouts. Furthermore, to guarantee objectivity and comparability, correction factors for more surfaces, such as clay or various indoor floorings, must be determined. Moreover, it is rational that different footwear [59] and physical capacities [60] have an impact on metabolic power that has not yet been investigated.

This systematic review has a few potential limitations. In line with all systematic reviews, selection bias regarding included studies cannot be completely precluded even though objectivity was improved as all studies were independently rated by two researchers. Additionally, because of the heterogeneity of the included studies, meaningful quantitative analyses such as a meta-analysis could not be implemented. However, as a compromise and strength of this systematic review, a best-evidence synthesis was conducted.

## Conclusions

In conclusion, this review shows that several validation studies for the metabolic power approach have been conducted over the last few years. However, especially the physiological validation studies were often partially implemented incorrectly as shown by the in-depth analysis of the underlying concept. Therefore, the described evidence levels should be treated with caution. Nevertheless, the approach is valid from a methodological point of view. Based on these findings and the modification of the concept during recent years, there is a need for further physiological validation studies. Therefore, it must be considered what the metabolic power approach can and cannot actually display. Moreover, there is a need to differentiate the approach in a sport, sub-group, and terrain specific manner as there are different metabolic demands and capacities.

## Data Availability

The datasets used and analyzed during the current study are available from the corresponding author on reasonable request.

## Abbreviations

ATP: Adenosine triphosphate
EC: Energy cost
EM: Equivalent mass
ES: Equivalent slope
GPS: Global positioning system
LPS: Local positioning system
P: Metabolic power
PRISMA: Preferred reporting items for systematic reviews and meta-analyses
